# Regurgitation and pulmonary aspiration during cardio-pulmonary resuscitation (CPR) with a laryngeal tube: A pilot crossover human cadaver study

**DOI:** 10.1371/journal.pone.0212704

**Published:** 2019-02-27

**Authors:** Kurt Ruetzler, Steve Leung, Mark Chmiela, Eva Rivas, Lukasz Szarpak, Sandeep Khanna, Guangmei Mao, Richard L. Drake, Daniel I. Sessler, Alparslan Turan

**Affiliations:** 1 Department of Outcomes Research, Anesthesiology Institute, Cleveland Clinic, Cleveland, Ohio, United States of America; 2 Department of General Anesthesiology, Anesthesiology Institute, Cleveland Clinic, Cleveland, Ohio, United States of America; 3 Department of Anesthesiology, Hospital Clinic, Barcelona, Spain; 4 Department of Emergency Medicine, University of Warszaw, Warszaw, Poland; 5 Department of Quantitative Health Sciences, Cleveland Clinic, Ohio, United States of America; 6 Department of Anatomy, Lerner College of Medicine at Cleveland Clinic, Cleveland, Ohio, United States of America; San Gerardo Hospital, ITALY

## Abstract

**Background:**

High-quality chest compressions are imperative for Cardio-Pulmonary-Resuscitation (CPR). International CPR guidelines advocate, that chest compressions should not be interrupted for ventilation once a patient’s trachea is intubated or a supraglottic-airway-device positioned. Supraglottic-airway-devices offer limited protection against pulmonary aspiration. Simultaneous chest compressions and positive pressure ventilation both increase intrathoracic pressure and potentially enhances the risk of pulmonary aspiration. The hypothesis was, that regurgitation and pulmonary aspiration is more common during continuous *versus* interrupted chest compressions in human cadavers ventilated with a laryngeal tube airway.

**Methods:**

Twenty suitable cadavers were included, and were positioned supine, the stomach was emptied, 500 ml of methylene-blue-solution instilled and laryngeal tube inserted. Cadavers were randomly assigned to: 1) continuous chest compressions; or, 2) interrupted chest compressions for ventilation breaths. After 14 minutes of the initial designated CPR strategy, pulmonary aspiration was assessed with a flexible bronchoscope. The methylene-blue-solution was replaced by 500 ml barium-sulfate radiopaque suspension. 14 minutes of CPR with the second designated ventilation strategy was performed. Pulmonary aspiration was then assessed with a conventional chest X-ray.

**Results:**

Two cadavers were excluded for technical reasons, leaving 18 cadavers for statistical analysis. Pulmonary aspiration was observed in 9 (50%) cadavers with continuous chest compressions, and 7 (39%) with interrupted chest compressions (P = 0.75).

**Conclusion:**

Our pilot study indicate, that incidence of pulmonary aspiration is generally high in patients undergoing CPR when a laryngeal tube is used for ventilation. Our study was not powered to identify potentially important differences in regurgitation or aspiration between ongoing vs. interrupted chest compression. Our results nonetheless suggest that interrupted chest compressions might better protect against pulmonary aspiration when a laryngeal tube is used for ventilation.

## Background

More than 350,000 people suffer out-of-hospital cardiac arrests annually in the United States.[1] Early initiation of advanced cardiopulmonary resuscitation (CPR) is key to favorable outcomes. Airway management is also crucial, and adequate ventilation and oxygenation is essential.[2]

Endotracheal intubation is generally considered the optimal method of managing the airway during CPR.[3, 4] An endotracheal tube promotes effective ventilation and oxygenation, minimizes gastric insufflation, and consequently reduces the risk of regurgitation and pulmonary aspiration.[5, 6] However, endotracheal intubation is challenging and requires considerable experience and skills, along with regular retraining.[7–9] Supraglottic airway devices are less invasive, much easier to insert than endotracheal intubation, and consequently reduce the time needed for successful intubation.[10–14] Supraglottic airway devices, including the laryngeal tube are thus increasingly used, especially by less experienced providers including paramedics.[15] A limitation common to all supraglottic airway devices is that they provide little protection against regurgitation and pulmonary aspiration[5, 16, 17]—and aspiration is a major clinical concern since it promotes pneumonia and acute respiratory distress syndrome.[18]

High quality chest compressions are the most important component during CPR, and even small interruptions much reduce systemic blood flow.[19] Consequently, international CPR guidelines stress that interruptions of chest compression should be limited to the extent possible; for example, even ventilation should be restricted to a 30:2 chest compression/ ventilation ratio until the airway is secured. They further specify that once a patient’s trachea has been intubated or a supraglottic airway device has been inserted, chest compression should not be interrupted for ventilation of the lungs.[4, 6, 20]

Simultaneous chest compressions and positive pressure ventilation, both increase intrathoracic pressure and potentially enhances the risk of regurgitation and pulmonary aspiration. However, it remains unknown whether continuing CPR during ventilation promotes regurgitation and pulmonary aspiration in patients ventilated through supraglottic airway devices. The aim of this pilot, cross-over study was therefore to evaluate the effect of continuing or interrupting chest compressions during ventilation on regurgitation and pulmonary aspiration. In particular, we considered the hypothesis that pulmonary aspiration is more common during continuous *versus* interrupted chest compressions in human cadavers ventilated with a laryngeal tube airway.

## Methods

Human cadavers were provided by the Department of Anatomy of the Cleveland Clinic Lerner College of Medicine. At the Cleveland Clinic the IRB has determined that since the cadavers used in research studies are donated through the Cleveland Clinic Body Donation Program the Director of that program, Dr. Richard Drake, will provide final approval for all studies using cadavers. There does not need to be a separate IRB approval.

We used human cadavers with a body mass index <45 kg/m^2^ without rigor mortis. All retained lifelike tissue characteristics and anatomical structures, as determined by an attending anatomist. Cadavers were warmed to ambient temperature before study.

Exclusion criteria’s included known pathologies of the upper airway or upper alimentary tract including oral cavity, pharynx, larynx, oesophagus and stomach. We also excluded cadavers that had previous surgery or radiation to the head, neck, chest, oesophagus, or stomach. And finally, we excluded cadavers that had a current or previous tracheostomy, or previous known regurgitation or aspiration of gastric content.

### Protocol

Suitable human cadavers were positioned supine on an autopsy table. The face, mouth, teeth, and upper aerodigestive tract were inspected to identify any injuries or lesions, along with evidence of previous regurgitation of gastric contents.

The belt of a CPR board Lucas (Physio Control, Redmond, WA) was properly mounted on the torso of the cadaver. The Lucas device uses a piston and suction cup to deliver chest compressions with active recoil. The system was correctly positioned by an experienced investigator, closely following the manufacturer’s instructions. Chest compression depth was set to 5 cm.

A conventional gastric tube was inserted orally and the stomach was emptied. Thereafter, 500 ml of 0.01% methylene-blue-solution (Merck Chemicals, Darmstadt, Germany) was instilled into the stomach and the tube removed. We selected 500 ml because larger volumes increase the risk of aspiration.[21] Fibreoptic bronchoscopy confirmed that there was no unwanted methylene-blue staining of the pharynx or larynx. The cadaver was then intubated using an appropriately sized laryngeal tube (King LTS-D, Ambu, Noblesville, IN, USA). Correct positioning, an acceptable seal, and good ventilation were confirmed by giving a single breath delivered with a bag-valve mask.

Each cadaver was evaluated under two conditions: 1) continuous chest compressions at a rate of 100/min with ventilation at a rate of 10 breaths/min (a breath every six seconds without pausing chest compressions); and, 2) interrupted chest compressions with ventilation at a rate of 30:2, with compressions paused as little as practical. Randomization (1:1) was based on computer-generated codes that were maintained in opaque envelopes until after intubation. Ventilation complied with current recommendations, with each breath lasting about 1 sec and having sufficient volume to slightly raise the chest.[22]

**Fig 1:**
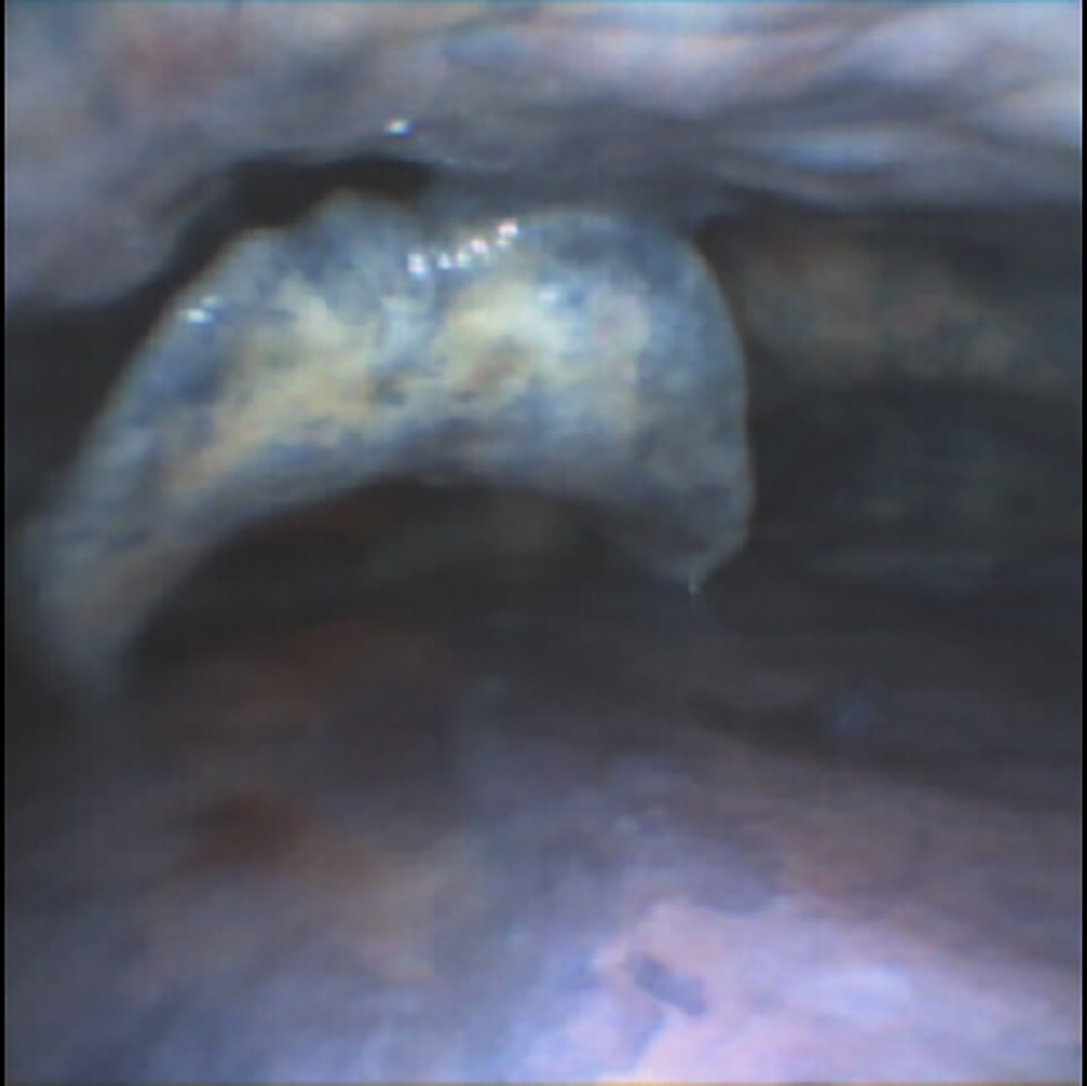
Regurgitation of methylene-blue solution, assessed by flexible bronchoscopy.

**Fig 2:**
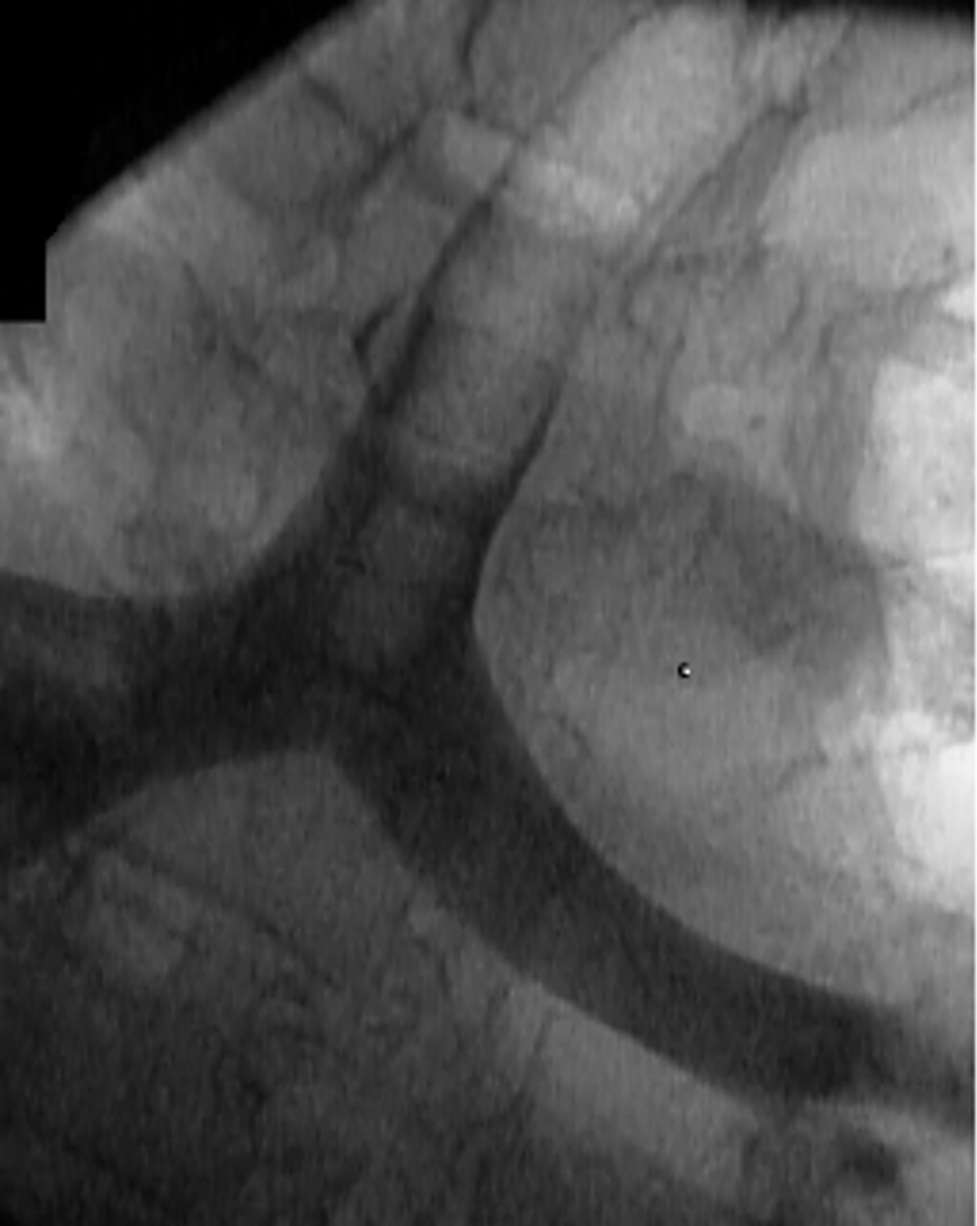
Pulmonary aspiration of barium sulfate radiopaque suspension, assessed by chest x-ray.

After 14 minutes of the initial designated CPR and ventilation, both were stopped and the amount of regurgitation and pulmonary aspiration were assessed using a conventional flexible bronchoscope (Fig 1). The dye solution was aspirated from the stomach and replaced with 500 ml of Liquid E-Z Paque, barium sulfate radiopaque suspension (Bracco Diagnostics Inc., Monroe Twp., NJ). Again, fiberoptic bronchoscopy was used to confirm that there was no contamination of the pharynx or larynx. CPR was then restarted with the designated ventilation strategy and maintained for another 14 minutes. Thereafter regurgitation and pulmonary aspiration was assessed with a conventional chest X-ray (Fig 2).

Oesophageal regurgitation was defined when methylene blue or barium sulfate was detected either by bronchoscopy or radiograph in the upper oesophagus, and/or larynx.[5] Pulmonary aspiration was defined by methylene blue or barium sulfate within the trachea below the vocal cords.[5]

### Statistical analysis

Given the *a priori* pilot nature of the project, we did not conduct a formal sample-size estimate. Instead, we planned to evaluate 20 cadavers because we expected that many to be available over a reasonable data-acquisition period. Our primary goal was to roughly estimate the incidence of pulmonary aspiration and regurgitation with each CPR and ventilation approach, thus providing information to guide the sample-size estimate of a potential future trial.

We estimated the incidences of pulmonary aspiration and regurgitation with 95% bootstrap confidence intervals for each group and the overall study population. Bootstrap confidence intervals were used instead of binomial confidence intervals to account for within-pair correlation. Additionally, we roughly estimated the effect of continuous (versus interrupted) compressions on pulmonary aspiration using a McNemar test for paired data.

## Results

We studied 20 cadavers, but two were excluded for technical reasons, one assigned to each initial treatment. Therefore 18 cavers were included in the statistical analyses, 9 starting with continuous chest compressions and 9 starting with interrupted chest compressions S1 Table. The characteristics of cadavers was summarized in **Table 1**. Overall, 7 (39%) were female; the mean of age was 66 (SD = 12) and the mean of BMI was 26 (SD = 6).

**Table 1:** Characteristics of cadavers.

	Overall	Randomization schedule	
Factor	(N = 18)	Continuous then interrupted (N = 9)	Interrupted then continuous (N = 9)
Age—years	66 ± 12	71 ± 12	62 ± 9
Gender—female %	7 (39%)	3 (33%)	4 (44%)
Body Mass Index–kg/m^2^	26 ± 6	27 ± 6	26 ± 6

Continuous variables were summarized as mean ±SD. Categorical variable was summarized as n (%).

Regurgitation was detected in all cadavers during continous compression, and 15 (83%) with interupted compression. Pulmonary aspiration was observed in 9 (50%) cadavers with continuous chest compressions, and 7 (39%) with interupted chest compression (P = 0.75). None of the differences was statistically significant (**Table 2**).

**Table 2:** Rate of regurgitation and aspiration.

	Chest compression	Rate (%)	95% CI*
**Aspiration**	Continuous	9 (50%)	(28%, 72%)
	Interrupted	7 (39%)	(17%, 61%)
**Regurgitation**	Continuous	18 (100%)	(100%, 100%)
	Interrupted	15 (83%)	(67%, 100%)

* 95% confidence intervals (CIs) were estimated from 1000 times bootstrapping.

## Discussion

Interruption of chest compressions decrease coronary perfusion pressure, reduce rate of return of spontaneous circulation (ROSC), diminish defibrillation success, and unsurprisingly are associated with poor outcome.[23] Consequently, interruptions should be kept as short as necessary and avoided when practical.[4, 6, 20] Several observational studies reported significant increases in survival rates among patients having CPR, at least in patients with a shockable rhythm.[24–26] although by far the largest prospective trial investigating the impact of continuous versus interrupted chest compression, failed to demonstrate any increase of survival and favorable neurologic function in patients suffering from out-of-hospital CPR.[27] Nonetheless, current CPR guidelines advocate continuous chest compressions once the airway is secured by an endotracheal tube or a supraglottic airway device.[4, 20]

Our study steamed from the suspicion that continuous chest compression might promote aspiration because of the simultaneous increases in intrathoracic pressure from chest compression and positive pressure ventilation, especially in patients ventilated through supraglottic airway devices. In our human cadaver pilot study, the strategy of continuous chest compressions and reducing the time without chest compressions, was associated with an absolute 10% increase in the incidence of pulmonary aspiration, although the difference was statistically not significant.

Passive regurgitation and pulmonary aspiration of gastric content is normally prevented by pressure from the distal esophagus sphincter and the muscle tonus of the esophagus. Both appear to be much reduced during CPR as suggested by the high incidence of pulmonary aspiration in the peri-CPR setting.

Aspiration was first described by *Mendelsohn* in 1946 and is defined as the inhalation of gastric contents (or oro-pharyngeal secretions) into the larynx and into the lower respiratory tract.[28] Clinical consequences of pulmonary aspiration varies and may often remain unrecognized, and without any significant clinical consequences. However, clinical deterioration mostly depends on the origin of the aspirate, and may be caused by chemical pneumonitis, infectious pneumonia, exacerbation of a pre-existing asthmatic disease, acute lung injury or even acute respiratory distress syndrome.[29] The incidence of pulmonary aspiration during CPR is extremely difficult to assess and is currently mostly unknown unclear, but estimates range from 20 to 65%.[18, 30, 31]

*Perbet and colleagues* report that 65% of patients given out-of-hospital CPR suffered from early-onset pneumonia,[30] although some pneumonia may have resulted from systemic inflammatory response syndrome or lung contusion.[30] In routine medico-legal autopsies, aspiration of gastric contents are observed in up to 25% of all cases (independent of the cause of death).[31] Furthermore, the timing of pulmonary aspiration in outpatients given CPR ranges from before CPR is initiated, during airway intervention (e.g. by a possible vomiting stimulus), during CPR after airway intervention (as in our study), or even thereafter during hospitalization.

The incidence of regurgitation is about 12% in patients who have in-hospital CPR, and 20% for out-of-hospital CPR.[16, 32] But in a previous human cadaver study, the incidence of regurgitation during CPR ranged between 0 and 80% depending on the airway device used.[5] In our current study, every human cadaver showed evidence of regurgitation during continuous chest compressions and nearly all (83%) did during interrupted chest compressions. The higher incidence in our human cadavers might be based on a relatively large 500-ml gastric volume and the prolonged CPR duration of 14 minutes. Regurgitation is concerning because it is required for aspiration but is not itself of any substantive clinical importance. In contrast, mortality from pulmonary aspiration ranges from 3.5 to 12%.[29]

We studied cadavers because they well approximate the anatomy and regurgitation risks in humans having CPR.[5] Consequently, cadavers are widely used in CPR studies and their findings are generally considered reliable. Animal or manikin models are almost surely less realistic.[33] A clinical trial would of course provide the best evidence, but it seems unlikely that one will address our question any time soon. To provide a close resemblance to live tissues, our cadavers were not preserved and each cadaver was prewarmed to ambient temperature. A limitation is that like most cadavers donated for medical research, ours came from elderly subjects and were at the lower end of the BMI spectrum. Results may have differed had we used cadavers with other characteristics, but it seems likely that the general sense of our finding would remain similar.

We did not measure the extent of pulmonary aspiration. However, clinical consequences mostly depend on the origin of the aspirate, instead of the extent. [29]

Another limitation of our trial is that we did not measure any ventilation minute volume and instead gave clinically reasonable breaths. Both hypoventilation and hyperventilation are associated with poor outcome after CPR.[34] Presumably aspiration risk increases as a function of both tidal volume and peak airway pressure.

In summary, our human cadaver pilot study was not powered to identify potentially important difference in regurgitation or aspiration. The results nonetheless suggest that interrupted chest compression might better protect against pulmonary aspiration when a laryngeal tube is used for ventilation. A well-powered trial is needed to confirm our observations.

## Supporting information

S1 Table Raw data used for analyses. (PDF)
