# Supplementary material for: Regurgitation and pulmonary aspiration during cardio-pulmonary resuscitation (CPR) with a laryngeal tube: A pilot crossover human cadaver study
Source: PLoS One. 2019 Feb 27;14(2):e0212704. doi: 10.1371/journal.pone.0212704 (PMC6392290; doi:10.1371/journal.pone.0212704)
Supplement: S1 Table — (PDF) [file pone.0212704.s001.pdf]

study_id	1st treat	gender	age	bmi	gp1_continuous_pharynx	gp1_continuous_aspiration	gp1_interrupted_pharynx	gp1_interrupted_aspiration
667-01-0000	Interrupted	0	52	17.49				
667-01-0001	Continuous	0	65	38.84	1	0	0	0
667-01-0002	Interrupted	1	55	30.3				
667-01-0003	Continuous	0	51	25.43	1	1	1	0
667-01-0004	Interrupted	0	60	24.35				
667-01-0005	Continuous	0	78	31.7	1	0	1	0
667-01-0006	Interrupted	0	79					
667-01-0007	Continuous	0	71	29.26	1	0	1	1
667-01-0008	Interrupted	0	60	21.51				
667-01-0009	Continuous	1	71	22.63	1	0	1	1
667-01-0010	Continuous	0	68	27.12	1	0	1	0
667-01-0011	Continuous	1	59	26.63	1	1	1	1
667-01-0012	Interrupted	1	73	25.38				
667-01-0013	Interrupted	0	60	22.31				
667-01-0014	Continuous	0	87	20.09	1	0	1	1
667-01-0015	Continuous	1	87	20.94	1	0	1	1
667-01-0016	Interrupted	1	52	28.32				
667-01-0017	Interrupted	1	63	34.87				

gp2_interrupted_pharynx	gp2_interrupted_aspiration	gp2_continuous_pharynx	gp2_continuous_aspiration
0	0	1	1
1	0	1	1
0	0	1	1
1	0	1	0
1	0	1	1
1	0	1	0
1	1	1	1
1	1	1	1
1	0	1	1
